# Molecular cloning and metabolomic characterization of the 5-enolpyruvylshikimate-3-phosphate synthase gene from *Baphicacanthus cusia*

**DOI:** 10.1186/s12870-019-2035-0

**Published:** 2019-11-09

**Authors:** Jian Yu, Yihan Zhang, Shuju Ning, Qi Ye, Hexin Tan, Ruibing Chen, Qitao Bu, Rui Zhang, Peimin Gong, Xiaoli Ma, Lei Zhang, Daozhi Wei

**Affiliations:** 1 College of Life Science, Fujian Agriculture and Forestry University, Fuzhou, 350002 People’s Republic of China; 2 Fujian Provincial Key Laboratory of Agroecological Processing and Safety Monitoring, Fuzhou, 350002 People’s Republic of China; 3 Key Laboratory of Crop Ecology and Molecular Physiology, Fuzhou, 350002 People’s Republic of China; 4 Department of Pharmaceutical Botany, School of Pharmacy, Second Military Medical University, Shanghai, 200433 People’s Republic of China; 5 School of Life Sciences, East China Normal University, Shanghai, 200433 People’s Republic of China; 6 Department of Pharmaceutical Changzheng Hosipital, Second Military Medical University, Shanghai, 200433 People’s Republic of China; 7 College of Crop Science, Fujian Agriculture and Forestry University, Fuzhou, 350002 People’s Republic of China

**Keywords:** Indigo alkaloids, *Baphicacanthus cusia*, 5-enolpyruvylshikimate-3-phosphate synthase, Molecular cloning, Enzyme assay, Plant metabolic engineering

## Abstract

**Background:**

Indigo alkaloids, such as indigo, indirubin and its derivatives, have been identified as effective antiviral compounds in *Baphicacanthus cusia*. Evidence suggests that the biosynthesis of indigo alkaloids in plants occurs via the shikimate pathway. The enzyme 5-enolpyruvylshikimate-3-phosphate synthase (EPSPS) is involved in plant metabolism; however, its underlying putative mechanism of regulating the production of indigo alkaloids is currently unknown.

**Results:**

One gene encoding EPSPS was isolated from *B. cusia*. Quantitative real-time PCR analysis revealed that *BcEPSPS* was expressed at the highest level in the stem and upregulated by methyl jasmonate (MeJA), salicylic acid (SA) and abscisic acid (ABA) treatment. The results of subcellular localization indicated that *BcEPSPS* is mainly expressed in both the plastids and cytosol, which has not been previously reported. An enzyme assay revealed that the heterogeneously expressed *BcEPSPS* protein catalysed the generation of 5-enolpyruvyl shikimate-3-phosphate. The overexpression of *BcEPSPS* in *Isatis indigotica* hairy roots resulted in the high accumulation of indigo alkaloids, such as indigo, secologanin, indole and isorhamnetin.

**Conclusions:**

The function of *BcEPSPS* in catalysing the production of EPSP and regulating indigo alkaloid biosynthesis was revealed, which provided a distinct view of plant metabolic engineering. Our findings have practical implications for understanding the effect of *BcEPSPS* on active compound biosynthesis in *B. cusia*.

## Background

*Baphicacanthus cusia (Nees) Bremek*, is widely distributed in Fujian, Yunnan, Sichuan and Guangdong provinces in China and is an important medicinal plant. Indigo naturalis (Qingdai) is made from its leaves and stems, which is known as “Jian Qingdai” and is a famous regional drug in Fujian Province. It is clinically used to treat leukaemia [1], oral cancer [2] and ulcerative colitis [3, 4]. *B. cusia* roots are used as a valuable drug named “Nan-Ban-Lan-Gen” [5]. To date, the main compounds isolated and identified from *B. cusia* are indole alkaloids [6], terpenoid alkaloids [7], quinoline ketone alkaloids [8], sterols, flavonoids [9], lignans [10], amino acids, organic acids [11], and plant polysaccharides [9]. Indole alkaloids are the main components of indigo naturalis and the main active constituents of the *B. cusia.*

The synthesis of indole alkaloids in *B. cusia* is generally considered to involve the shikimate pathway and the indole pathway [12–14], which was based on the knowledge of microbial indigo synthesis. However, the biosynthetic pathway of the indole alkaloids in *B. cusia* remains unknown. Therefore, we propose a hypothetical biosynthetic pathway of indole alkaloids in vivo according to the microbial synthesis pathway (Additional file 3: Figure S2C).

The enzyme 5-enolpyruvylshikimate-3-phosphate synthase (EPSPS; EC 2.5.1.19) catalyses the transfer of enolpyruvyl moiety of phosphoenol pyruvate (PEP) to 5-hydroxyl shikimate-3- phosphate (S3P) (Additional file 3: Figure S2B), producing shikimic acid and chorismite, which then enters the biosynthetic pathway of indole alkaloids. Therefore, EPSPS is the first key enzyme in this biosynthetic pathway. Recent research has established that glyphosate acts as a competitive inhibitor relative to PEP and binds adjacent to S3P in the active-site of EPSPS; additionally glyphosate maintains the regulatory function of *PtrEPSPS* in the phenylpropanoid pathway of *Populus* [15–18]. However, there are limited studies on the function of *BcEPSPS* in the metabolic pathway, In the context of overexpression, purification and kinetic characterization of *BcEPSPS*, this paper will provide a theoretical and experimental basis for explaining the biological synthesis pathway and molecular regulation mechanism of the active compounds in *B. cusia*.

## Results

### Isolation and characterization of *BcEPSPS*

**Fig. 1:**
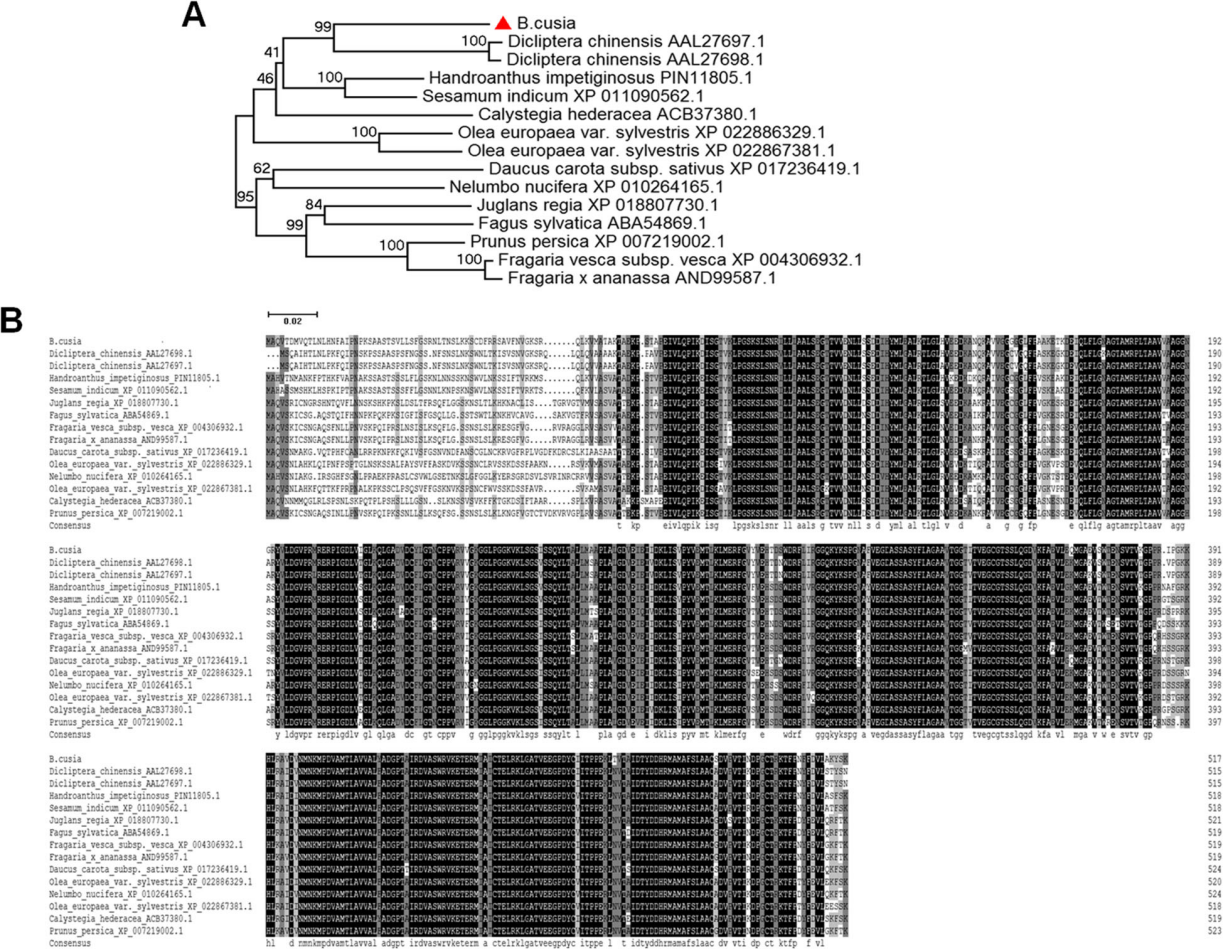
Bioinformatics analysis of *BcEPSPS*. **a** Phylogenetic tree of the EPSPS protein family from 14 species of plants using MEGA 5.0 software by the neighbour-joining method. **b** The homology comparison of amino acid alignment of EPSPS in 14 plant species

The cDNA of *BcEPSPS* contains an open reading frame of 1554 nucleotides, and is translated into an amino acid sequence of 518 aa with a calculated molecular mass of 55.33 kDa. The isoelectric point (pI) is 7.55, which illustrated that the *BcEPSPS* protein is slightly basic, and the protein has an obvious hydrophobicity area of − 0.295 and a hydrophilicity area (Additional file 2: Figure S1D). The *BcEPSPS* protein has a stable structure, which includes no signal peptide, and a small portion of the trans-membrane topological structure may exist in its secondary structure (Additional file 2: Figure S1E, F). The main secondary structures of the *BcEPSPS* protein are predicted to involve 42.86% random coil, 32.82% alpha helix, 6.56% beta turn and 17.76% extended strand. Random coils and alpha helices were the most abundant structural elements distributed throughout most parts of the *BcEPSPS* secondary structure, while beta turns and extend strands were intermittently distributed in the protein (Additional file 2: Figure S1B). It also contains two main structural domain: the EPSPS-synthase active structural domains and the PLN02338 conserved domain (Additional file 2: Figure S1A). The 3D structure of *BcEPSPS* was predicted and simulated by SWISS-MODEL and phyre2 using sequence homology-based structural modelling (PDB id: 3nvc.1.A) with the consistency of predicted results at 57.35% (Additional file 2: Figure S1G), which showed that it contains the 3-phosphoshikimate-1-carboxyvinyltransferase functional domain. Ramachandran conformation also showed that *BcEPSPS* has a stable space conformation (Additional file 2: Figure S1H). A phylogenetic tree was constructed for the EPSPS protein family by comparing *B. cusia* with 13 other species of plants by phylogenetic analysis, which indicated that EPSPS in *Dicliptera chinensis* appeared to be phylogenetically in the same clade as *BcEPSPS* (Fig. 1a). The homology comparison of the amino acid sequences indicated that *B. cusia* shared a high homology with several plants such as *Handroanthus impetiginosa*, *Sesamum indicum*, and *Calystegia hederacea* (Fig. 1b).

### Expression and induction patterns of *BcEPSPS*

**Fig. 2:**
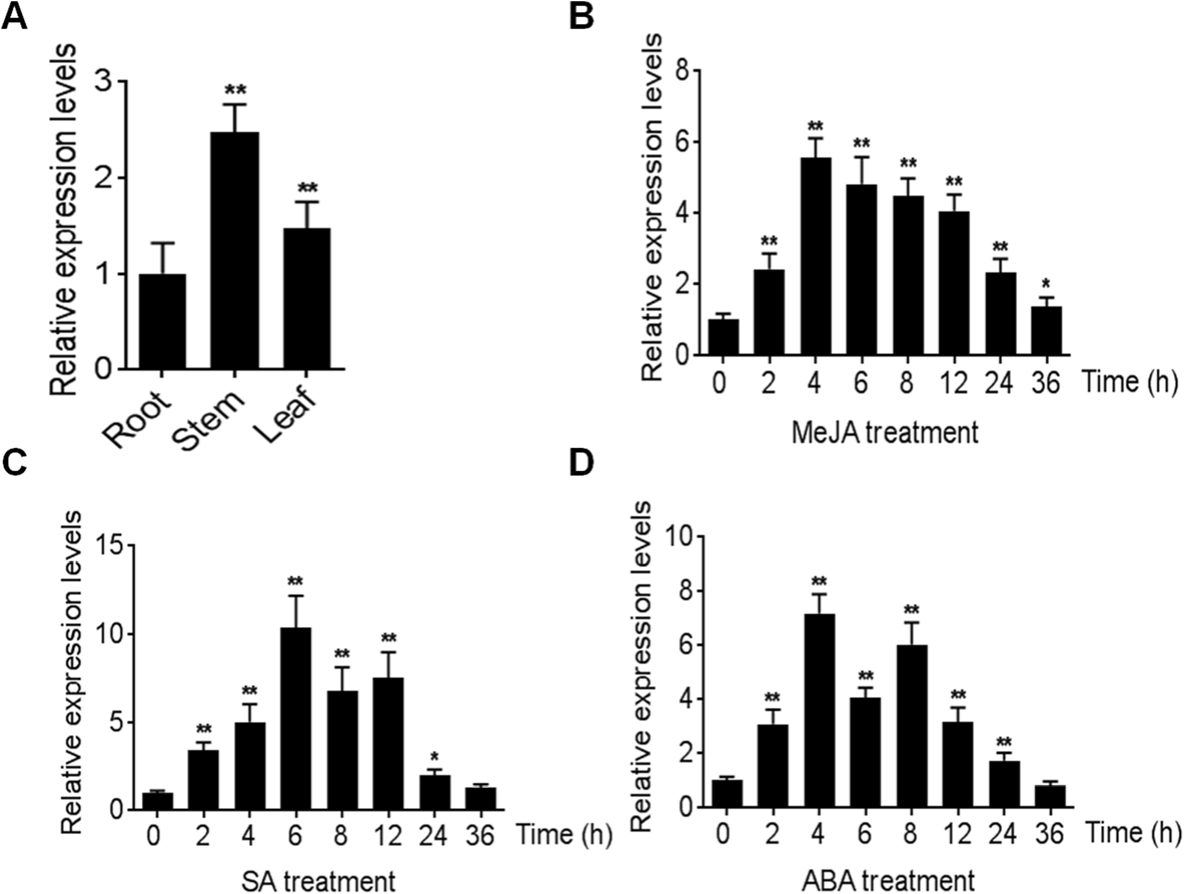
*BcEPSPS* expression profiles in *B.cusia*. **a** The expression profiles of *BcEPSPS* in different *B.cusia* tissues, and under phytohormone induction (MeJA (**b**), SA (**c**), ABA (**d**)). Data are represented as the mean ± SEM; *P < 0.05 and, **P < 0.01 compared to the control group

To analyse the expression patterns of *BcEPSPS*, total RNA was isolated from the roots, stems and leaves of *B. cusia* and qPCR was performed. The results showed constitutive expression of *BcEPSPS* in different tissues, with maximum expression in the stem followed by the leaves and the lowest expression in the roots (Fig. 2a). qRT-PCR results showed that *BcEPSPS* was upregulated by MeJA, SA and ABA, and the expression levels showed obvious variations related to time and phytohormones (Fig. 2b, c, d). For MeJA treatment, the expression level of *BcEPSPS* rapidly reached a maximum at 4 h, with a 5.79-fold increase and subsequently declined. After treatment with SA, the expression level of *BcEPSPS* reached the highest level at 6 h (10.65-fold), followed by a short decline and a slight increase until 12 h, then fell to the initial levels. In response to ABA treatment, the expression level of *BcEPSPS* increased quickly and reached the peak at 4 h which was 7.96-fold higher than that of the control. Then, the expression level fell first and rose again during the 4–8 h period, but it dropped below the initial level at 36 h.

### Subcellular localization of *BcEPSPS*

**Fig. 3:**
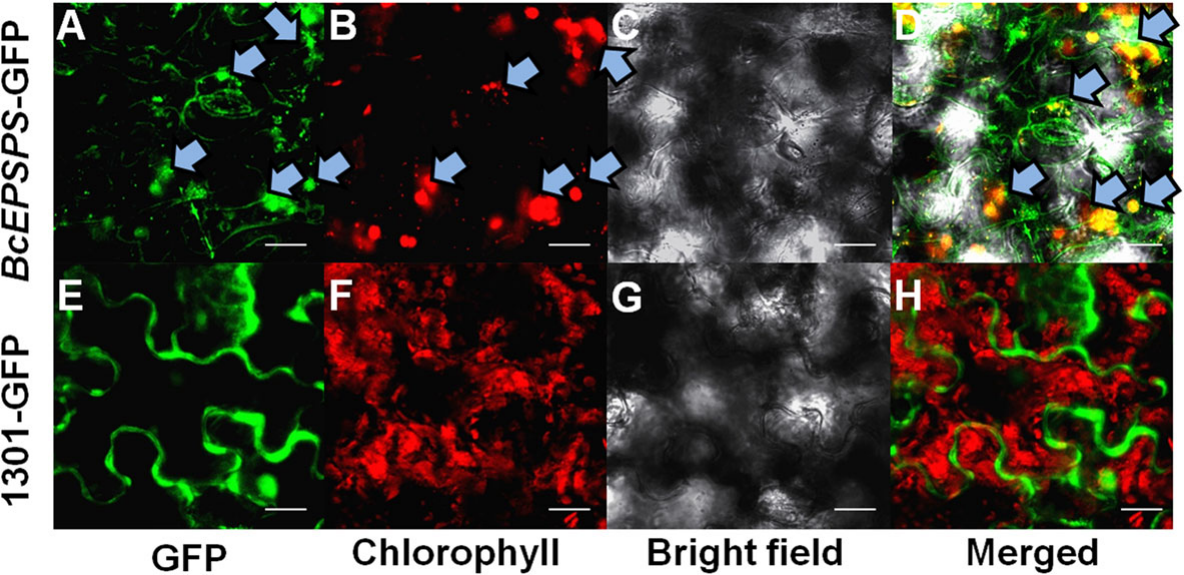
Subcellular localization of *BcEPSPS*. **a** *I. indigotica* cells expressing *BcEPSPS*-GFP showed green fluorescent signals mainly in the chloroplast and slightly in the cytoplasm. **b** The same nucleus and cytoplasm showing the chlorophyll autofluorescence signal in the cell. **c** Bright-field image. **d** The merged signal of panels **a** and **b**. **e**) *I. indigotica* cells expressing GFP showed green fluorescent signals in the cytoplasm. **f** The same cell showing the chlorophyll auto fluorescence signal in the cytoplasm. **g** Bright-field image. (**h**) The merged signal of panels E and F. Bars = 5 nm

To further verify the expression characteristics of *BcEPSPS*, we examined the subcellular localization of *BcEPSPS* in the leaves of *N.tabacum*, with a control that included an empty *pCAMBIA* 1301-GFP vector (Fig. 3), Interestingly, the *BcEPSPS*-GFP fusion protein was localized in both the plastids and cytosol (Fig. 3a-d). In contrast, the *pCAMBIA* 1301-GFP vector showed florescence throughout the entire cell (Fig. 3e-h).

### The expression and identification of the recombinant *BcEPSPS* protein

**Fig. 4:**
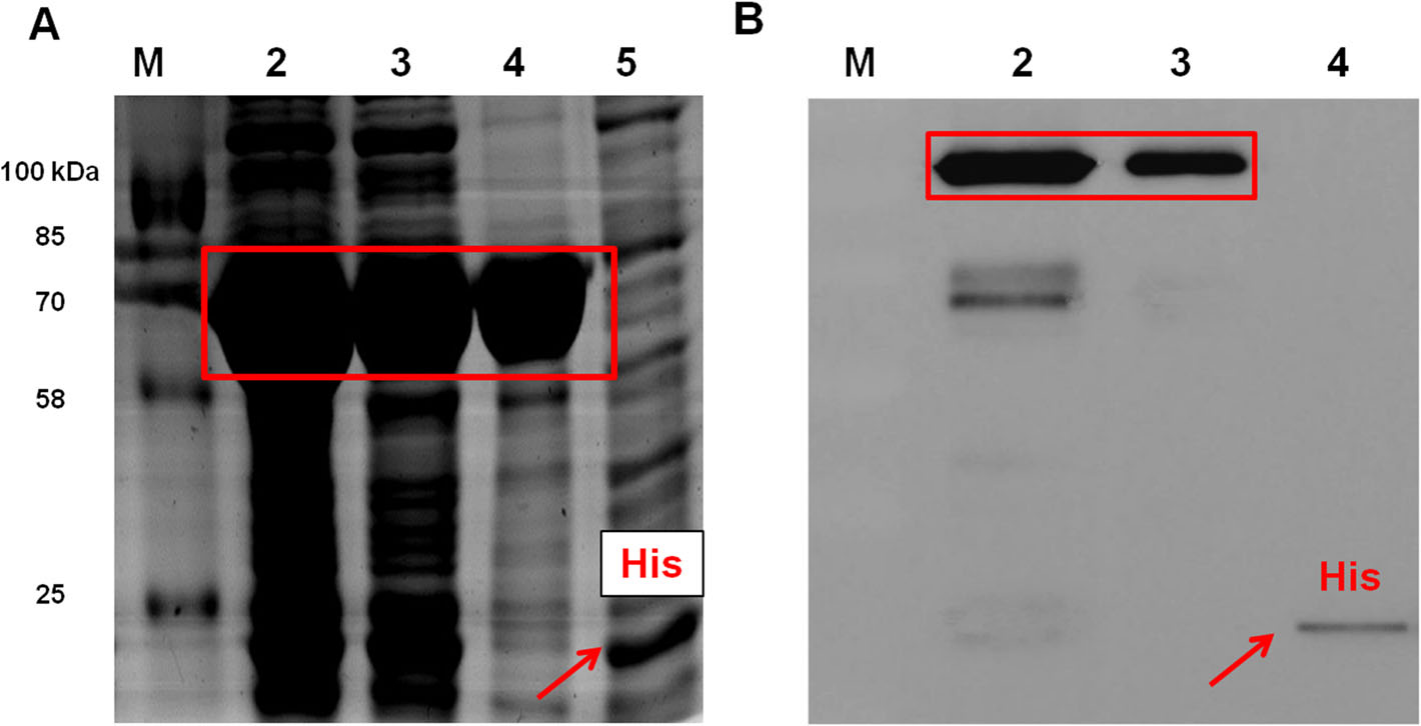
Functional characterization of *BcEPSPS* in vitro. **a** SDS-PAGE analysis of *BcEPSPS* purified by Ni-NTA conjugation. Lane 1, protein molecular weight marker; lane 2, crude enzyme extract from *BcEPSPS*-pET-32a cell lysate; lane 3, supernatant of pET-32a-*BcEPSPS* cell lysate; lane 4, purified pET-32a-*BcEPSPS* protein; lane 5, crude enzyme extract from pET-32a cell lysate. **b** Western blotting analyses of *BcEPSPS*. Lane 1, protein molecular weight marker; lane 2, supernatant of pET-32a-*BcEPSPS* cell lysate; lane 3, purified *BcEPSPS* protein; lane 4, purified pET-32a protein

**Fig. 5:**
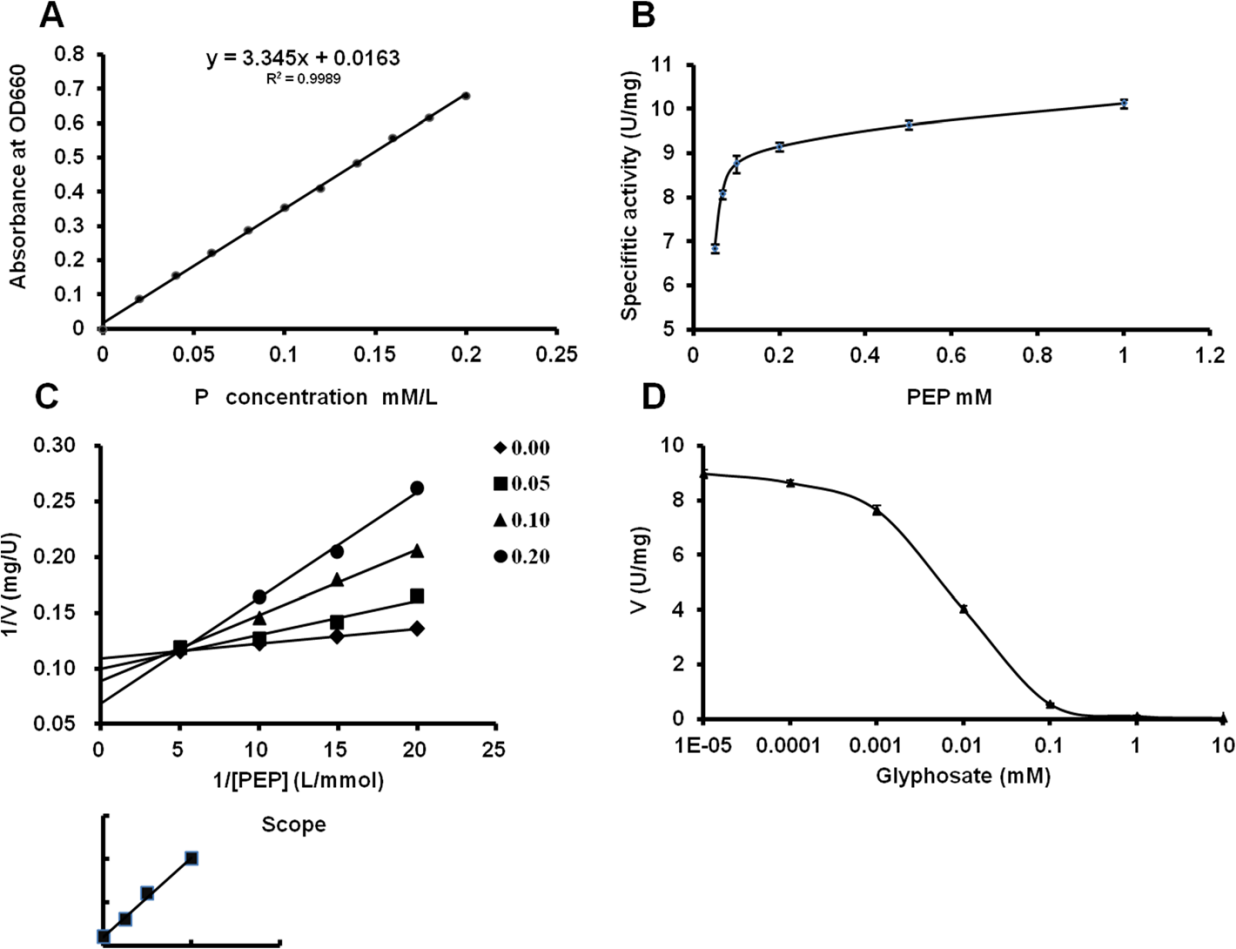
Enzyme kinetic characterizations of *BcEPSPS* in vitro. **a** Calibration curve of inorganic phosphorus concentration. **b** *Km*_(PEP)_curve. **c** *Ki*_(glyphosate)_curve. **d** *IC*_*50*_ curve

To test the function of *BcEPSPS*, the *BcEPSPS* recombinant protein was purified through prokaryotic expression and *BcEPSPS* enzyme activity was determined in vitro. The molecular weights of *BcEPSPS* and His-tag were ~ 55 kDa and ~ 15 kDa respectively, and the fusion protein *BcEPSPS*-His was approximately 70 kDa, as predicted (Fig. 4a). Figure 5a shows the fusion protein *BcEPSPS*-His induced by IPTG (1 mM), and the purified *BcEPSPS*-His protein showed a single distinct band in lane 4. The protein concentration was determined using the Bradford method. The standard curve equation was Y = 0.8726x + 0.0623, R^2^ = 0.9981, and the concentration of purified recombinant *BcEPSPS*-His protein was calculated as 7.96 mg/mL. The result of Western blotting indicated that purified recombinant *BcEPSPS*-His protein exhibited anti-His antibody immune reactivity (Fig. 4b).

### The kinetic properties of purified *BcEPSPS*

Purified *BcEPSPS* was subjected to enzymatic activity assays under various conditions. As shown in Fig. 5, the Pi standard curve was conducted (Fig. 5a). Then, we measured the velocity of the *BcEPSPS* catalytic reaction under the constant of S3P at 1.0 mM and the increasing constant of PEP, where the *Km*_(PEP)_ was 21.267 μM and Vmax was 10.259 U/mg (Fig. 5b). To test *Ki*_(glyphosate)_, the constant of S3P was 1.0 mM, and the concentration of glyphosate was set as increasing constants. This showed that *Ki*_(glyphosate)_ was 0.046 μM (Fig. 5c). For IC_50_, the velocity was measured under various concentrations of PEP (0.05, 0.067, 0.1, and 0.2 mM), while the concentration of glyphosate was set as 10^− 5^, 10^− 4^, 10^− 3^, 10^− 2^, 10^− 1^, 1 and 10 mM. Figure 5d shows that the IC_50_ was 6.37 μM, which indicates that the *BcEPSPS* protein is type I and is sensitive to glyphosate. Moreover, the kinetic properties of the *BcEPSPS* protein are represented in Table 1.

**Table 1:** Kinetic properties of purified recombinant *BcEPSPS*

Kinetic constants	*BcEPSPS*
Enzymatic	10.11 ± 0.76
IC_50_ (μM)	6.37
*Km*_(PEP)_ (μM)	21.267 ± 0.98
*Ki*_(glyphosate)_ (μM)	0.046
Vmax (U/mg)	10.259 ± 0.038
*Ki/Km*_(PEP)_	0.00216

### Improvement in the indigo alkaloid contents by the overexpression of *BcEPSPS* in *I. indigotica*

**Fig. 6:**
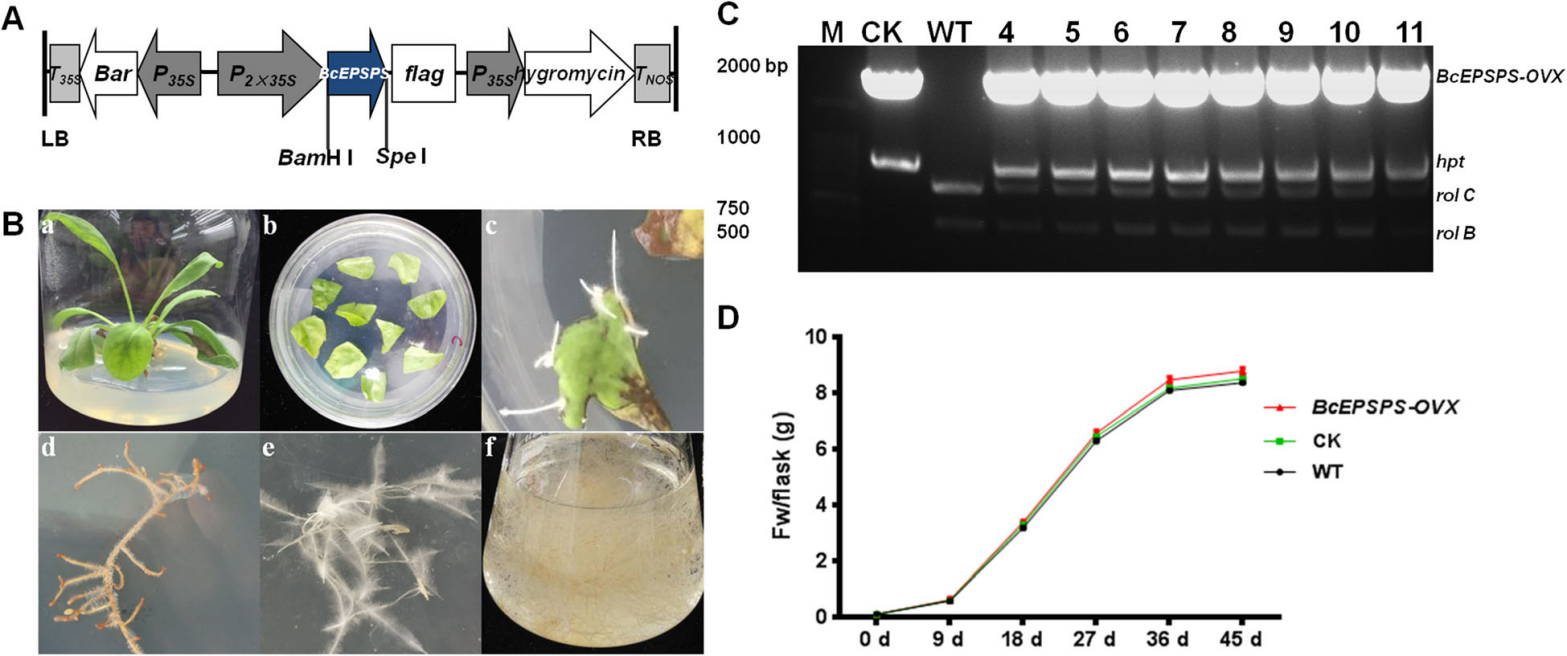
Hairy roots of *OVX-BcEPSPS* in *I. indigotica*. (**a**) Schematic representation of the *BcEPSPS* overexpression vector. T35S, Bar, P35S, CaMV 35S promoter, flag tag, P35S, hygromycin resistance gene. (**b**) Induction and culture of *I. indigotica* hairy roots in different periods. a, A sterilised plant of *I. indigotica*. b, *Agrobacterium tumefaciens* C58C1 stains carrying plasmids were applied to infect the sterilized leaf. c, The hairy roots were derived from sterile leaves after 2 weeks. d, The false positive hairy roots without the vector C58C1-*OVX-BcEPSPS* on the 1/2 MS solid medium were supplemented with a gradual decrease in cefotaxime and hygromycin. e, The positive hairy roots containing the vector C58C1-*OVX-BcEPSPS* showed great growth on the 1/2 MS solid medium with cefotaxime and hygromycin. f, Liquid cultures of positive hairy roots containing the vector C58C1-*OVX-BcEPSPS* in an Erlenmeyer flask in 45 days. (**c**) Molecular identification of transgenic hairy roots. Lane 1, DNA size marker, lane 2, the engineered strain as the positive control, lane 3, wild-type control. Lane 4–11, strains of *OVX-BcEPSPS*. The primers *hpt*, *rolb* and *rolc* were used to check the hygromycin resistance and plasmid *Ri*. (**d**) Growth rates of WT, CK and *OVX-BcEPSPS* hairy root lines

*BcEPSPS* driven by the double CaMV 35S promoter in the overexpression vector PHB-flag was introduced into *I. indigotica* to generate *OVX-BcEPSPS* hairy root cultures by *Agrobacterium* C58C1. The vector PHB-flag and *Agrobacterium* C58C1 were individually transferred into *I. indigotica* to generate the control cultures and the wild-type cultures. *I. indigotica* hairy roots were induced and cultured for different periods (Fig. 6b). PCR analysis demonstrated that the exogenous *BcEPSPS* gene was integrated into the transgenic lines, which contained the *rolb*, *rolc* and hygromycin resistance (*hyg*) genes (Fig. 6c). The growth rates of the hairy root lines are shown in Fig. 6d. The *OVX-BcEPSPS* hairy root lines exhibited a more vigorous growth rate than the Con and CK lines.

**Fig. 7:**
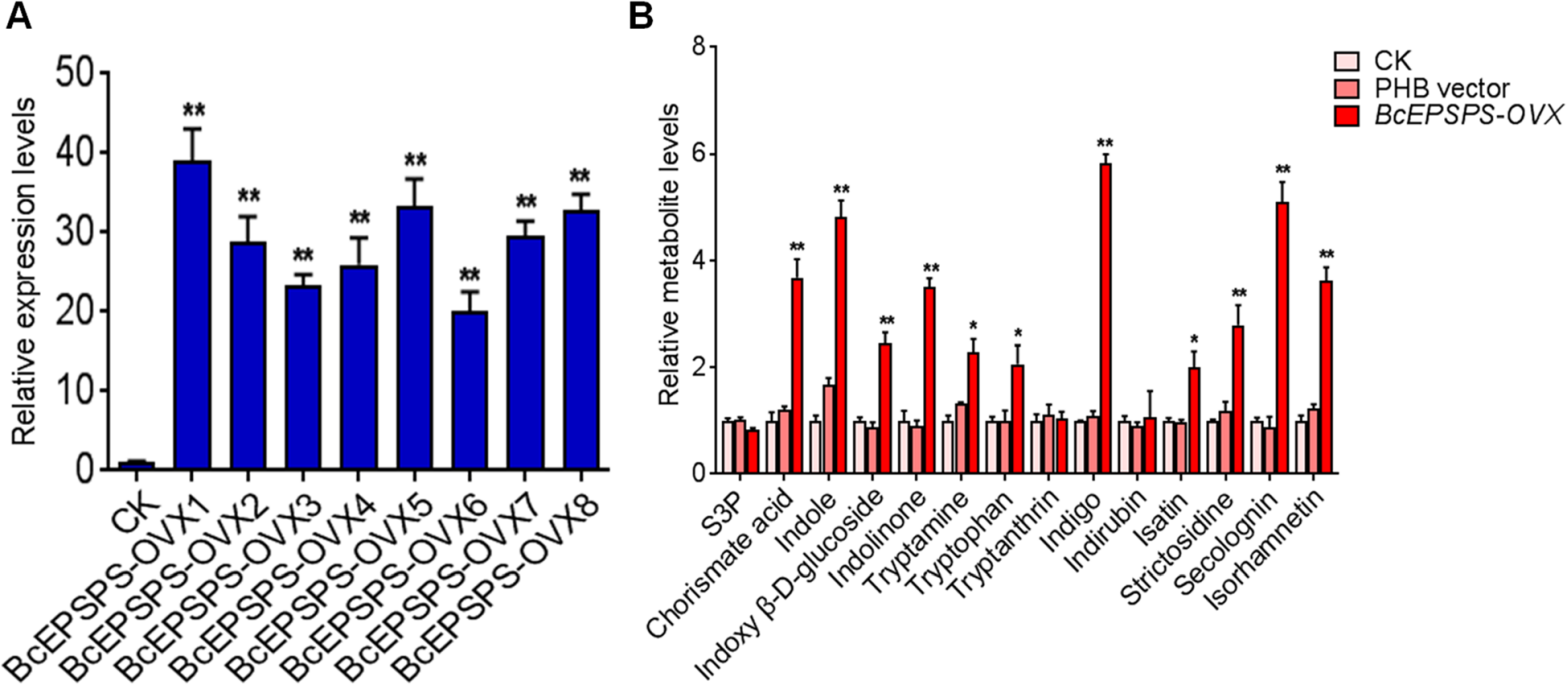
Metabolite analysis of transgenic hairy roots. **a** *BcEPSPS* transcript abundance in *OVX-BcEPSPS* transgenic hairy roots by RT-qPCR. **b** Relative metabolite levels in C58C1, PHB vector and OVX-*BcEPSPS* transgenic hairy roots. Data are represented as the mean ± SEM; *P < 0.05 and, **P < 0.01 compared to the control group

QRT-PCR analysis was performed to examine the transcript levels of the endogenous *BcEPSPS* in positive lines. In comparison to the expression level of the WT controls, the expression level of *BcEPSPS* in the *OVX-BcEPSPS* lines was significantly enhanced by 37.87-fold (Fig. 7a). Metabolite analysis indicated that the contents of active compounds such as indigo, secologanin, indole, isorhamnetin, chorismic acid, strictosidine and indolinone were improved in varying degrees in transgenic lines compared with the WT lines and the CK lines. The chromatograms with corresponding retention times of 14 chemical compounds are shown in Additional file 4: Figure S3, in which the detection of signals and the degree of separation is distinct and intuitive. The indigo and secologanin accumulation in the *OVX-BcEPSPS* lines were significantly higher (5.83- and 5.09-fold) than those in the control lines. Additionally, the indole, isorhamnetin, strictosidine and indole beta-D-glucoside contents were 4.82-, 3.61-, 2.79- and 2.78-fold higher respectively in the *OVX-BcEPSPS* lines than the WT and CK lines (Fig. 7b). There were no significant differences between the WT and CK lines (Fig. 7b).

## Discussion

EPSP synthases are known to have the unitary function of catalysing shikimate-3-phosphate to 5-enolpyruvylshikimate-3-phosphate in the shikimate pathway in microorganisms and plants [19, 20]. In plants, the shikimate pathway is upstream of the indole pathway and provides chorismic acid for the biosynthesis of indole alkaloid compounds [20]. Here, we provide evidence that EPSP synthase plays an important regulatory role in the accumulation of indole alkaloids and terpenoid alkaloids in *B. cusia*.

In this research, the 5-enolpyruvylshikimate-3-phosphate synthase gene was isolated and identified from the data of the *B. cusia* transcriptome, and the characteristics of cDNA sequences were predicted through bioinformatic software. Tissue-specific expression results showed that the *BcEPSPS* genes were expressed at the highest level in stem tissues, consistent with a recent study, in which the expression level of the *BcSK* gene in *B. cusia* under MeJA treatment increased with increasing time [20]. The qPCR results indicated that the *BcEPSPS* level was upregulated by MeJA, SA and ABA, with variations in the induction time and expression level. Our subcellular localization results demonstrated that the *BcEPSPS* protein did not accumulate in the plastids but did accumulate in the cytosol, a finding that has not been previously reported. Moreover, a recent study on PtrEPSP-TF protein from *Populus*, revealed its expression in the nucleus [18]. Although the direct mechanism underlying the observed cytosol presence remains to be determined, one explanation is that the *BcEPSPS* protein might interact with transcription factors. Our enzymatic activity assay results demonstrated that the ectopically expressed and purified *BcEPSPS* protein has catalytic activity in vitro. Additionally, *BcEPSPS* in *B. cusia* is sensitive to glyphosate, which indicates that the *BcEPSPS* protein is a class I protein. As several glyphosate-resistant mutants of class I EPSPS have been identified, and most of the class I EPSPS mutants display increased glyphosate resistance [21], it is useful to create a glyphosate-resistant mutant of a *B. cusia* EPSPS using a directed evolution strategy [22].

Alkaloids are orgainc compounds that contain nitrogen, and are important, effective components of Chinese herbal medicine with significant biological activities [23–25]. Indole alkaloids are a class of alkaloids identified by the presence of a structural moiety of indole [26]. The levels of indole alkaloids in *B. cusia* are higher than that those in other plants such as *I. indigotica*, *Polygonum tinctorium* and *Indigofera tinctoria*, and there was an obvious difference in the contents of indoxyl beta-D-glycoside, indigo and indirubin between different organs [27]. A gene overexpression vector was built for hairy roots, and was transferred by *Agrobacterium* C58C1. The metabolite analysis indicated that the levels of active compounds such as indigo, secologanin, isorhamnetin and strictosidine were improved to varying degrees in the transgenic lines compared with the controls. Finally, the molecular catalytic characteristics of *BcEPSPS* in indole alkaloid biosynthesis in *B. cusia* reported here will provide a unique perspective for functional studies of the shikimate pathway.

The development of functional genomics technologies and genetic transformation technologies has greatly facilitated research on alkaloid biosynthesis [28–30]. Additionally, next-generation sequencing provides a fast, high-throughput approach for medicinal plants, for which current genetic information is limited, and is used to obtain batch sequences. Such data will provide a new perspective of molecular research on *B. cusia*, for which the main active substances are indole alkaloids, and will aid in protecting germplasm resources of *B. cusia* by sorting and revealing functional genes.

## Conclusions

The enzyme 5-enolpyruvylshikimate-3-phosphate synthase is involved in catalysing indole alkaloid biosynthesis. *BcEPSPS* was first isolated and identified from the data of the *B. cusia* transcriptome, and the function and characteristics of *BcEPSPS* were further verified in vitro. High concentrations of indole alkaloids were produced in the hairy root cultures of *BcEPSPS* overexpression. It has been suggested that through the regulation of *BcEPSPS*, the accumulation of effective components can be improved.

In conclusion, a hairy root overexpression culture system with a high concentration of indoline accumulation was established in this study, and it is expected to alleviate the over-exploitation and shortage of high quality resources of *B. cusia* in the future.

## Methods

### Plant materials and treatments

Samples of *B. cusia* were obtained from the experimental field of Xianyou county in Putian city, China. Species verification was performed by Professor Daozhi Wei of the College of Life Science, Fujian Agriculture and Forestry University (FAFU). These samples were preserved in the laboratory of medicinal plant resources in FAFU (No.004512-ML). *I. indigotica* plants were cultivated in the experimental field of the Second Military Medical University (SMMU), Shanghai, China. Species verification was performed by Professor Lei Zhang of the School of Pharmacy, SMMU. These samples were preserved in the laboratory of medicinal plant resources in SMMU (No. S1011078). The sterilized seedlings of *I. indigotica* were cultured on Murashige and Skoog (MS) medium solidified with 0.6% agar. The organ-specific series of samples (root, stem and leaf, 6 months old) were used for RNA extraction. Leaves of the *B. cusia* were sprayed with 0.1 mM methyl jasmonate (MeJA), salicylic acid (SA), and abscisic acid (ABA) for stress treatments [31], and leaf samples were harvested at 0, 2, 4, 6, 8, 12, 24 and 36 h post-treatment. Three independent biological replicates for each group were performed. MeJA, SA and ABA were purchased from Sigma-Aldrich (USA). All samples were immediately frozen in liquid N_2_ and stored at − 80 °C for further research.

### Total RNA isolation and first-strand cDNA synthesis

Total RNA of *B. cusia* plants was extracted using the TransZol Up Plus RNA Kit (TransGen Biotech). One microgram of RNA was used to prepare first strand cDNA using the TransScript First-Strand cDNA Synthesis Super Mix (TransGen Biotech). The concentration and quality of RNA and first-strand cDNA samples were examined by spectrophotometer analysis using a NanoDrop 2000C Spectrophotometer (Thermo) and agarose gel electrophoresis with ethidium bromide.

### Isolation and characterization of *BcEPSPS*

The *BcEPSPS* gene sequence was isolated from a transcriptome database of *B. cusia*, with the gene-specific primers EPSPS-F and EPSPS-R (Additional file 1: Table. S1). The full-length coding region of *BcEPSPS* was cloned by PCR with KD Plus DNA Polymerase (TransGen Biotech) (Additional file 5: Figure S4). PCR was performed under the following conditions: denaturation at 94 °C for 5 min, 35 cycles of 94 °C for 30 s, 55 °C for 30 s and 68 °C for 2 min, and a final extension at 68 °C for 10 min. After gel electrophoresis detection, the PCR products were purified and cloned into a pBlunt-Zero vector (TransGen Biotech) and transformed into Trans1-T1 cells (TransGen Biotech) for sequencing.

### Bioinformatics analysis of *BcEPSPS*

The open reading frame of *BcEPSPS* was searched and translated on the NCBI ORF Finder (http://www.ncbi.nlm.nibi.gov/gorf/gorf.htmL). The composition and physicochemical properties of *BcEPSPS* were calculated with Protparam (http://www.expasy.ch/tools/protparam.htmL). The hydrophobicity/hydrophilicity of the *BcEPSPS* amino acid sequence was predicted with ProtScale (http://www.expasy.ch/tools/protscale.htmL). The signal peptide and leading peptide of the *BcEPSPS* protein were analysed on Signal 3.0 (http://www.cbs.dtu.dk/services/Signal/) and TargetP 1.1 (http://www.cbs.dtu.dk/services/TargetP/) respectively. The prediction of the transmembrane domains and coiled-coils of the *BcEPSPS* protein were accomplished with TMPred (http://www.ch.embnet.org/software/TMPRED_form.htmL) and COILS (http://www.ch.embnet.org/software/COILS_form.htmL) respectively. The secondary structure and functional domains of the *BcEPSPS* protein were detected the SOPMA (http://npsapbil.ibcp.fr/cgibin/npsa_automat.pl/page=/NPSA/npsa_sopma.htmL), Pfam24.0 (http://www.Pfam.sanger.ac.uk/search) and BLAST (http://www.ncbi.nlm.nibi.gov/Structure/cdd/wrpsb.cgi). The model of the three-dimensional structure and Ramachandran conformation were completed on Swiss-Model (http://www.expasy.ch/swissmod/SWISS-MODEL.htmL) and PyMOL respectively. The homology comparison and phylogenetic tree were generated by MEGA5.0.

### Quantitative real-time PCR (qPCR)

qPCR analysis was performed to determine the expression features of *BcEPSPS* and was conducted on a Thermal Cycler Dice Real Time PCR machine (TaKaRa, Japan), using the TransStart Top Green qPCR SuperMix Kit (TransGen Biotech) according to the manufacturer’s instructions. The first strand cDNA was generated by the TransScript One-Step gDNA Removal and cDNA Synthesis SuperMix (TransGen Biotech), by adding Oligo (dT) primer, gRemover, E-mix, and R-mix to 1 μg of total RNA and the manufacturer’s protocol was followed. qPCR was performed by using the 2-^ΔΔ^Ct method under the following conditions: denaturation at 94 °C for 30 s, followed by 45 cycles of 94 °C for 5 s and 60 °C for 30 s, followed by a final dissociation stage. The expression levels were normalized with the 18S housekeeping gene [32]. Primers for qPCR are listed in Additional file 1: Table S1.

### Subcellular localization in the cells of *Nicotiana tabacum* leaves

To confirm the subcellular localization of *BcEPSPS*, the encoding region of *BcEPSPS* was cloned into the *pCAMBIA1301-GFP* vector (Additional file 6: Figure S5). The primers for constructing the vector are listed in Additional file 1: Table S1. Vectors *BcEPSPS-GFP* and GFP were transiently expressed in *N.tabacum* (20 days old) for the observation of subcellular localization. The plants were incubated for 24 h in darkness and the leaf sections were observed using a confocal laser scanning microscope (Nikon, Japan) [33].

### Purification and activity assays of the *BcEPSPS* protein

The encoding region of *BcEPSPS* was cloned into pET-32a (Novagen) (Additional file 7: Figure S6) and expressed in *E. coli* strain BL21. The primers for constructing the vector and for sequencing are listed in Additional file 1: Table S1. The cells were grown overnight at 37 °C in 200 mL of Luria-Bertani broth containing 75 μg/L ampicillin to an OD600 of 0.6, and the cells were further induced with 1 mM isopropylthio-b-galactoside at 16 °C for 24 h. After sonication lysis and centrifugation - the supernatant of the extraction buffer containing the crude extracted enzyme was purified at 4 °C using nickel-chelate affinity chromatography (Bio-Rad Laboratories, USA). The protein concentration was measured with the Bradford Protein Assay Kit (Sangon). The extraction buffer containing the purified enzyme was added to glycerin to 10% (w/v) and stored at − 80 °C. Western blotting was used to determine the induced expression of His-tagged EPSPS with an anti-rabbit mAb His antibody (Cell Signaling Technology), and a secondary antibody (Anti-rabbit IgG, HRP-linked Antibody).

### Analysis of enzyme activity

*BcEPSPS* activity was determined by measuring the release of inorganic phosphate in each reaction using the malachite green dye assay method [34]. The reaction was assayed in 100 μl of 50 mM HEPES/NaOH (pH 7.0), 1 mM PEP, 1 mM S3P, and 0.75 μg of purified enzyme. After incubation at 30 °C for 3 min, 800 μl of malachite green/ammonium molybdate colorimetric solution was added. One min later, the colour reaction was stopped by adding 800 μl (34%) sodium citrate solution. The absorbance at 660 nm was measured after incubation at room temperature for 0.5 h [35, 36].

Kinetic characterization was performed in a reaction buffer containing different concentrations of PEP or glyphosate. The kinetic data were fit to the appropriate equations using the program SigmaPlot (SPSS Science, Chicago, 1 L). The *Km* value was generated by fitting the data to V = Vmax [S]/(*Km* + [S]), where V is the velocity of the reaction (U/mg), Vmax is the maximum velocity, *Km* is the Michaelis constant and [S] is the concentration of the substrate. The *Ki* value was determined by a previously described method [37]. The S3P concentration was fixed at 1 mM, and the glyphosate concentration was fixed at 0, 0.05, 0.1 and 0.2 μM. The IC_50_ of enzyme activity was determined by fitting the data to *V* = *Vmin* + (*Vmax* – *Vmin*)/(1 + ([I]/IC_50_)s), where [I] is the concentration of glyphosate, s is the slope of the curve at IC_50_ [38], and *V* was determined at 1 mM PEP and S3P with different glyphosate concentrations ranging from 0.00001 mM to 10 mM.

### Generation and analysis of *BcEPSPS* overexpression in hairy roots

The encoding region of *BcEPSPS* was cloned into binary vector PHB-flag, which is used as an overexpression vector with two CaMV 35S promoters [39] (Fig. 6a) (Additional file 8: Figure S7). The primers for constructing the vector are listed in Additional file 1: Table S1. To induce *I. indigotica* hairy roots, *Agrobacterium tumefaciens* strain C58C1 (containing a *Ti* plasmid) with plasmids *BcEPSPS*-PHB and PHB (control check, CK) were applied to infect the sterilized leaf [40, 41]. The infected leaves were placed on 1/2 MS solid medium supplemented with 500 mg·mL^− 1^ cefotaxime. After 2 weeks, the transgenic hairy roots were derived from sterile leaves that were approximately 2–3 cm long. The hairy roots were then excised and cultured on 1/2 MS solid medium supplemented with 500 mg·mL^− 1^ cefotaxime and 10 mg·mL^− 1^ hygromycin. The 1/2 MS solid medium was replaced every 2 weeks with a gradually decreasing concentration of cefotaxime (300, 200,100 0 mg·mL^− 1^). *BcEPSPS*-PHB lines were labelled *OVX-EPSPS* and the blank C58C1 line was labelled wild-type (WT). Approximately 100 mg of the transgenic hairy roots and WT lines were cultivated in 250 mL Erlenmeyer flasks containing 200 mL of fresh liquid 1/2 MS medium supplemented with 10 mg mL^− 1^ hygromycin on an orbital shaker maintained at 110 rpm and 25 °C in darkness.

The hairy roots were subcultured every 9 days and harvested at 45 days for DNA and RNA extraction and metabolite analysis. Additionally, the fresh weights of the hairy roots were recorded at days 0, 9, 18, 27 and 45 to determine the biomass growth rate.

### Analysis of *BcEPSPS*-PHB in hairy roots

Genomic DNA of positive transgenic hairy roots was extracted using the Plant Genomic DNA Kit (TransGen Biotech), with primers for detection listed in Additional file 1: Table S1. Primer *Rbcsr* was used for testing the presence of *BcEPSPS*, while primers *hpt*, *rolb* and *rolc* were used for checking the hygromycin resistance and plasmid *Ri*.

qRT-PCR was performed to analyse the expression of *BcEPSPS* in positive transgenic hairy roots. The expression levels were normalized with the *Actin* housekeeping gene [42].

### Extraction and determination of indigo alkaloids

Positive transgenic hairy roots were collected and dried at 40 °C for 2 days and ground into a fine powder. Two hundred milligrams of dry power was extracted with 5 mL of methanol and trichloromethane (1:1) via sonication for 1 h, the supernatant was transferred and the powder was extracted with 5 mL of reagents again. The supernatant was pooled and filtered through a 0.22 μm organic membrane. Five millilitres of the extracting solution was evaporated to dryness and redissolved in 200 μl methanol.

HPLC analysis was performed on an Agilent Technologies 1260 infinity (Agilent, USA) [43]. The mobile phase was 0.1% solution of formic acid (A phase) and 0.1% formic acid in methanol (B phase) (HPLC grade). The solvent gradient programme used was as follows: 0–15 min, 40% A and 60% B; 15–20 min, 60% A and 40% B; 20–30 min, 80% A and 95% B. The reaction monitoring mode for HPLC analysis involved the following conditions: Diamonsil C-18 (4.6 μm × 250 mm, 5 μm), a flow rate of 1.0 mL/min, a column temperature of 25 °C and an injection volume of 10 μL. All standards were purchased from Sigma-Aldrich (St. Louis, MO).

### Statistical analysis

Experiments were performed in triplicate, and all results were expressed as the mean ± SEM. Statistical analysis was performed with SPSS 13.0 software. A Student’s t-test was used to compare two groups. One-way ANOVA followed by the Dunnett post hoc test was used for multiple comparisons versus the control group (GraphPad Software). *p* < 0.05 and *p* < 0.01 were set as the criterion for statistical significance.

## Supplementary information

**Additional file 1: Table S1.** PCR primers used in the text.

**Additional file 2: Figure S1.** Bioinformatics analysis of *BcEPSPS*. (A) Prediction of the domains of the *BcEPSPS* protein. (B) Prediction of the secondary structure of the *BcEPSPS* protein. (C) Coiled-coil prediction of the *BcEPSPS* protein. (D) Prediction of hydrophobic/hydrophilic regions of the *BcEPSPS* protein. (E) Prediction of transmembrane domain of the *BcEPSPS* protein. (F) Signal peptide prediction for the *BcEPSPS* protein. (G) Model of the three-dimensional structure of the *BcEPSPS* protein. (H) The Ramachandran conformation of the *BcEPSPS* protein.

**Additional file 3: Figure S2.** Prediction of the biosynthetic pathway of effective components in *B. cusia* and the catalytic reaction of EPSPS. (A) The chemical structure of indigo and indirubin. (B) The catalytic reaction of EPSPS. (C) The pink area is the Shikimate pathway. DAHPS, 3-deoxy-D-arabino-heptulosonate 7-phosphate synthase. EPSPS, 5-enolpyruvylshikimate-3-phosphate synthase. CS, chorismate synthase. The grey part is the tryptophan and indole pathway. AS, anthranilate synthase. TSA, tryptophan synthase alpha. CYP450, cytochrome P450 monooxygenase. The green part is the mevalonate pathway. DXS, deoxy-D-xylulose-5-phosphate synthase. HDS, hydroxymethylbutenyl-4-diphosphate synthase. HMBD, 1-hydroxy-2-methyl-2(D)butenyl-4-diphosphate. IPK, isopentenyl pyrophosphate kinase. IPP, isopentenyl pyrophosphate. The orange part is the MEP/DOXP pathway. AACT, acetoacetyl coenzyme thiolase. MPK, mevalonate phosphate kinase. MDC, mevalonate diphosphate decarboxylase. STR, strictosidine synthase.

**Additional file 4: Figure S3.** Chromatograms of 14 chemical compounds. The chromatograms with corresponding retention times of 14 chemical compounds, shikimate-3-phosphate, chorismic acid, indole, indoxyl beta D-glucoside, indolinone, indigo, indirubin, isatin, tryptophan, tryptamine, trytanthrin, isorhamnetin, secologanin and strictosidine.

**Additional file 5.** The nucleotides and amino acid sequence of BcEPSPS.

**Additional file 6.** The pCAMBIA 1301-GFP vector

**Additional file 7.** The pET 32a vector for constructing the fusion protein BcEPSPS-His.

**Additional file 8.** The overexpression vector PHB-flag.

## Data Availability

The datasets used and/or analysed during the current study are included in this article and its additional files.

## Abbreviations

ABA: abscisic acid
AS: Anthranilate synthase
CS: Chorismate synthase
CYP450: Cytochrome P450 monooxygenase
DAHPS: 3-deoxy-D-arabino-heptulosonate 7-phosphate synthase
dw: Dry weight
DXS: Deoxy-D-xylulose − 5-phosphate synthase
EPSPS: 5-enolpyruvylshikimate-3-phosphate synthase
FAFU: Fujian Agriculture and Forestry University
fw: Fresh weight
HDS: Hydroxymethylbutenyl-4-diphosphate synthase
HMBD: 1-hydroxy-2-methyl-2(D)butenyl-4-diphosphate
IPK: Isopentenyl pyrophosphate kinase
IPP: Isopentenyl pyrophosphate
MDC: Mevalonate diphosphate decarboxylase
MeJA: Methyl jasmonate
MPK: Mevalonate phosphate kinase
qRT-PCR: Real-time quantitative PCR
SA: Salicylic acid
SARS: Severe acute respiratory syndromes
SMMU: Second Military Medical University
STR: Strictosidine synthase
UGT: UDP-sugar-dependent glycosyltransferase

## References

[1] Hu XM, Tanaka S, Onda K, Yuan B, Toyoda H, Ma R, Liu F, Hirano T (2014). Arsenic disulfide induced apoptosis and concurrently promoted erythroid differentiation in cytokine-dependent myelodysplastic syndrome-progressed leukemia cell line F-36p with complex karyotype including monosomy 7. Chin J Inter Med.

[2] Lo WY, Chang NW (2013). An indirubin derivative, indirubin-3′-monoxime suppresses oral cancer tumorigenesis through the down regulation of survivin. PLoS One.

[3] Suzuki H, Kaneko T, Mizokami Y, Narasaka T, Endo S, Matsui H, Yanaka A, Hirayama A, Hyodo I (2013). Therapeutic efficacy of the Qing Dai in patients with intractable ulcerative colitis. World J Gastroentero.

[4] Fan H, Liu XX, Zhang LJ, Hu H, Tang Q, Duan XY, Zhong M, Shou ZX (2014). Intervention effects of QRZSLXF, a Chinese medicinal herb recipe, on the DOR-β-arrestin1-Bcl2 signal transduction pathway in a rat model of ulcerative colitis. J Ethnopharmacol.

[5] Chinese Pharmacopoeia Commission: Pharmacopoeia of People’s Republic of China (I). Chin Medical Technology Press. 2015.

[6] Yang XX, Lv SH, Wu SJ (1995). Rearches on leaves from *Baphicacanthus cusia*. Chin Herbal Med..

[7] Li L, Liang HQ, Liao SX, Qiao CZ, Yang GJ, Dong TY (1993). Chemical studies of *Strobilanthes cusia*. Acta Pharm Sin.

[8] Honda G, Tabata M (1979). Isolation of antifungal principle tryptanthrin, from *Strobilanthes cusia* O. Kuntze. Planta Med.

[9] Wu YQ, Zhu HJ, Wang YS, Qian B, Zhang RP, Zou C. Rearches on chemical components in *Baphicacanthus cusia*. Chin Herbal Med. 2005:982–3.

[10] Wei HH, Wu P, Wei XY, Ji TYZ, Xie HH (2005). Study on glycosides in ban-Lan-gen. J Tropical Subtropical Plants.

[11] Liao FH (2003). Analysis on amino acids of Nan-ban-Lan-GEN. Chin J Veterinary Med.

[12] Maugard T, Enaud E, Sayette ADL, Choisy P, Legoy MD (2002). β-Glucosidase-catalyzed hydrolysis of Indican from leaves of *Polygonum tinctorium*. Biotechnol Prog.

[13] Warzecha H, Frank A, Peer M, Gillam EMJ, Guengerich FP, Unger M (2007). Formation of the indigo precursor indican in genetically engineered tobacco plants and cell cultures. Plant Biotechnol J.

[14] Dubouzet JG, Matsuda F, Ishihara A, Miyagawa H, Wakasa K (2013). Production of indole alkaloids by metabolic engineering of the tryptophan pathway in rice. Plant Biotechnol J.

[15] Dillon A, Varanasi VK, Danilova TV, Koo DH, Nakka S, Peterson DE, Tranel PJ, Friebe B, Gill BS, Jugulam M (2017). Physical mapping of amplified copies of the 5-enolpyruvylshikimate-3-phosphate synthase gene in glyphosate-resistant *amaranthus tuberculatus*. Plant Physiol.

[16] Sutton KA, Breen J, Russo TA, Schultz LW, UmLand TC (2016). Crystal structure of 5-enolpyruvylshikimate-3-phosphate (EPSP) synthase from the ESKAPE pathogen Acinetobacter baumannii. Acta Crystallogr.

[17] Filiz E, Koc I (2016). Genome-wide identification and comparative analysis of EPSPS (aroA) genes in different plant species. J Plant Biochem Biotech.

[18] Xie M, Wellington M, Anthony CB, Kelsey Y, Guo HB, Zhang J, Timothy JT, Vasanth RS, Erika L, Raja SP, Jaime BR, Richard D, Nancy E, Robert WS, Mark D, Sara SJ, Lee EG, Olivia T, Stephen PD, Luke ME, Kim W, Cassandra C, Jeremy S, Hong G, Udaya K, Miguel R, Feng K, Chen JG, Gerald AT (2018). A 5-enolpyruvylshikimate 3-phosphate synthase functions as a transcriptional repressor in *Populus*. Plant Cell.

[19] Maeda H, Dudareva N (2012). The shikimate pathway and aromatic amino Acid biosynthesis in plants. Ann Review Plant Biol.

[20] Mir R, Jallu S, Singh TP (2015). The shikimate pathway: review of amino acid sequence, function and three-dimensional structures of the enzymes. Crit Rev Microbiol.

[21] Huang Y, Tan H, Yu J, Chen Y, Guo Z, Wang G, Zhang Q, Chen J, Zhang L, Diao Y (2017). Stable internal reference genes for normalizing real-time quantitative PCR in *Baphicacanthus cusia* under hormonal stimuli and UV irradiation, and in different plant organs. Front Plant Sci.

[22] Frances HA, Patrick LW, Kentaro M, Anne G (2001). How enzymes adapt: lessons from directed evolution. Trends Biochem Sci.

[23] Aniszewski T (2007). Alkaloids-secrets of life. alkaloid chemistry, biological significance, applications and ecological role.

[24] Ziegler J, Facchini PJ (2008). Alkaloid biosynthesis: metabolism and trafficking. Annu Rev Plant Biol.

[25] Kingston DG (2009). Tubulin-interactive natural products as anticancer agents. J Nat Prod.

[26] Seigler DS (2006). Plant secondary metabolism. World Sci-Tech R & D.

[27] Liau BC, Jong TT, Lee MR, Chen SS (2007). LC-APCI-MS method for detection and analysis of tryptanthrin, indigo, and indirubin in Daqingye and Banlangen. J Pharmaceut Biomed Analysis.

[28] Farrow SC, Hagel JM, Facchini PJ (2012). Transcript and metabolite profiling in cell cultures of 18 plant species that produce benzylisoquinoline alkaloids. Phtochemistry..

[29] Pistelli L, Giovannini A, Ruffoni B, Bertoli A, Pistelli L (2010). Hairy roots cultures for secondary metabolites production. Adv Exp Med Biol.

[30] Scheible WR, Morcuende R, Czechowski T, Fritz C, Osuna D, Palacios-Rojas N, Schindelasch D, Thimm O, Udvardi MK, Stitt M (2004). Genome-wide reprogramming of primary and secondary metabolism, protein synthesis, cellular growth processes, and the regulatory infrastructure of arabidopsis in response to nitrogen. Plant Physiol.

[31] Ma RF, Xiao Y, Lv ZY, Tan HX, Chen RB, Li Q, Chen JF, Wang Y, Yin J, Zhang L, Chen WS (2017). AP2/ERF transcription factor, Ii049, positively regulates Lignan biosynthesis in *Isatis indigotica* through activating salicylic acid signaling and Lignan/lignin pathway genes. Front Plant Sci.

[32] Li T, Wang J, Lu M, Zhang T, Qu X, Wang Z. Selection and Validation of Appropriate Reference Genes for qRT-PCR Analysis in Isatis indigotica Fort. Front Plant Sci. 2017. 10.3389/fpls.2017.01139 PMC5487591 28702046

[33] You J, Zong W, Li X, Ning J, Hu H, Li X, Xiao J, Xiong L (2013). The SNAC1-targeted gene OsSRO1c modulates stomatal closure and oxidative stress tolerance by regulating hydrogen peroxide in rice. J Exp Bot.

[34] Lanzetta PA, Alvarez LJ, Reinach PS, Candia OA (1979). An improved assay for nanomole amounts of inorganic phosphate. Anal Biochem.

[35] He M, Yang ZY, Nie YF, Wang J, Xu P (2001). A new type of class I bacterial 5-enopyruvylshikimate-3-phosphate synthase mutants with enhanced tolerance to glyphosate. Biochim Biophys Acta.

[36] Zhou M, Xu H, Wei X, Ye Z, Wei L, Gong W, Wang Y, Zhu Z (2006). Identification of a glyphosate-resistant mutant of rice 5-enolpyruvylshikimate 3-phosphate synthase using a directed evolution strategy. Plant Physiol.

[37] Enzymes: A practical introduction to structure, mechanism, and data analysis.: RA Copeland, ed. VCH Publishers, Inc, Department of Book Review, VCH, Weinheim, 1996. Biomed Pharmacother*.* 1997, 51(4):187.

[38] Eschenburg S, Healy ML, Priestman MA, Lushington GH, Schönbrunn E (2002). How the mutation glycine96 to alanine confers glyphosate insensitivity to 5-enolpyruvyl shikimate-3-phosphate synthase from *Escherichia coli*. Planta..

[39] Masani MY, Parveez GK, Izawati AM, Lan CP, Siti ANA (2009). Construction of PHB and PHBV multiple-gene vectors driven by an oil palm leaf-specific promoter. Plasmid.

[40] Chen J, Xin D, Li Q, Xun Z, Gao S, Chen R, Sun L, Lei Z, Chen W (2013). Biosynthesis of the active compounds of *Isatis indigotica* based on transcriptome sequencing and metabolites profiling. BMC Genomics.

[41] Xiao Y, Qian J, Gao S, Tan H, Chen R, Li Q, Chen J, Yang Y, Zhang L, Wang Z (2015). Combined transcriptome and metabolite profiling reveals that IiPLR1 plays an important role in lariciresinol accumulation in *Isatis indigotica*. J Exp Bot.

[42] Vandesompele J, Preter KD, Pattyn F, Poppe B, Roy NV, Paepe AD, Speleman F (2002). Accurate normalization of real-time quantitative RT-PCR data by geometric averaging of multiple internal control genes. Genme Biol.

[43] Yang Y, Pu RY, Feng G, Zhang L, Lu WQ, Gao SH (2018). Simultaneous determination of four constituents in roots, stem and leaves from *Baphicacanthus cusia* by RP-HPLC and evaluation of three different drying methods. Chin J Hospital Pharmacy.

