# Supplementary material for: Molecular cloning and metabolomic characterization of the 5-enolpyruvylshikimate-3-phosphate synthase gene from Baphicacanthus cusia
Source: BMC Plant Biol. 2019 Nov 9;19:485. doi: 10.1186/s12870-019-2035-0 (PMC6842527; doi:10.1186/s12870-019-2035-0)
Supplement: Supplementary file 1 — Additional file 1: Table S1. PCR primers used in the text. [file 12870_2019_2035_MOESM1_ESM.docx]

**Table S1.** PCR primers used in the text

| **Primer name** | **Sequence** |
| --- | --- |
| *BcEPSPS*-ORF-F | ATGGCCCAAGTTACGGATATG |
| *BcEPSPS*-ORF-R | GTGTTTTGAATACTTGGCGAG |
| *BcEPSPS*-QPCR-F | TTACCAAACCCATTCGCTGC |
| *BcEPSPS*-QPCR-R | TTCTGGGTGGAACTGGACTC |
| *BcEPSPS*-sub-F | GGAAGATCTTATGGCCCAAGTTACGGATAT |
| *BcEPSPS*-sub-R | CGGACTAGTGTGTTTTGAATACTTGGCGAG |
| *BcEPSPS*-pET-F | CGCGGATCCCATGGCCCAAGT |
| *BcEPSPS*-pET-R | CCGCTCGAGGTGTTTTGAAT |
| S-Tag | CGAACGCCAGCACATGGACA |
| T7-Term | GCTAGTTATTGCTCAGCGG |
| *BcEPSPS*-ovx-F | AAAGGATCCATGGCCCAAGTTACGGATAT |
| *BcEPSPS*-ovx-R | AAAACTAGTGTGTTTTGAATACTTGGCGAG |
| *Rolb*-F | CGAGGGGATCCGATTTGCTT |
| *Rolb*-R | GACGCCCTCCTCGCCTTCCT |
| *Rolc*-F | TCGCCATGCCTCACCAACTCAC |
| *Rolc*-R | CCTTGATCGAGCCGGGTGAGAA |
| *hpt*-F | CGATTTGTGTACGCCCGACAGTC |
| *hpt*-R | CGATGTAGGAGGGCGTGGATATG |
| *Rbcsr* | ATTAACTTCGGTCATTAGAGGC |
| *Actin*-F | ATCCTCCGTCTTGACCTTGCT |
| *Actin*-R | TTTCCCGTTCTGCTGTTGTG |
| *18S*-F | GCTTCCCTCCCGACAATTTC |
| *18S-*R | AGTCGGGTTGTTTGGGAATG |
