# Supplementary figures and images for: Molecular cloning and metabolomic characterization of the 5-enolpyruvylshikimate-3-phosphate synthase gene from Baphicacanthus cusia
Source: BMC Plant Biol. 2019 Nov 9;19:485. doi: 10.1186/s12870-019-2035-0 (PMC6842527; doi:10.1186/s12870-019-2035-0)

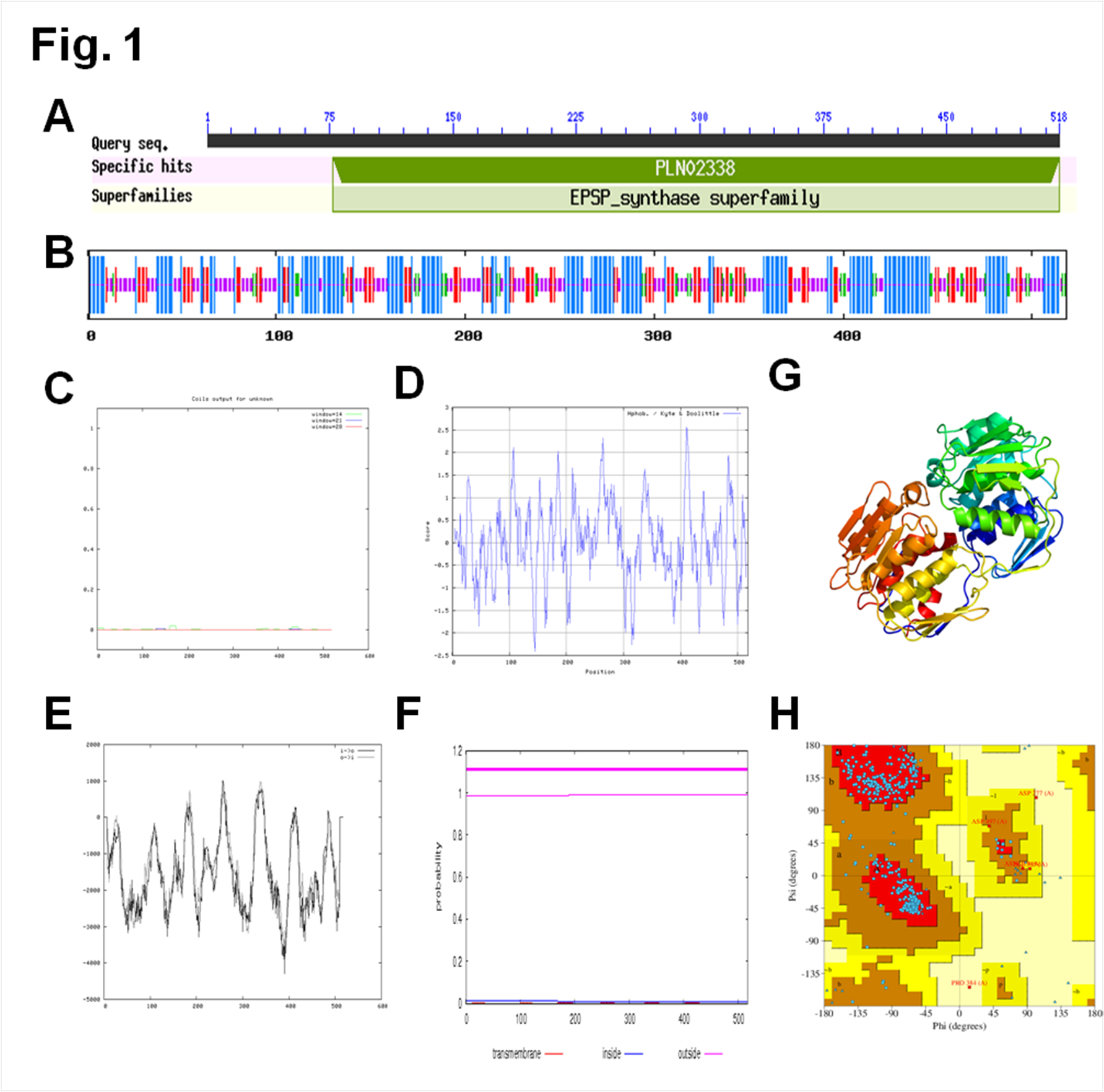

Supplement: Supplementary file 2 — Additional file 2: Figure S1. Bioinformatics analysis of BcEPSPS. (A) Prediction of the domains of the BcEPSPS protein. (B) Prediction of the secondary structure of the BcEPSPS protein. (C) Coiled-coil prediction of the BcEPSPS protein. (D) Prediction of hydrophobic/hydrophilic regions of the BcEPSPS protein. (E) Prediction of transmembrane domain of the BcEPSPS protein. (F) Signal peptide prediction for the BcEPSPS protein. (G) Model of the three-dimensional structure of the BcEPSPS protein. (H) The Ramachandran conformation of the BcEPSPS protein. [file 12870_2019_2035_MOESM2_ESM.tif]

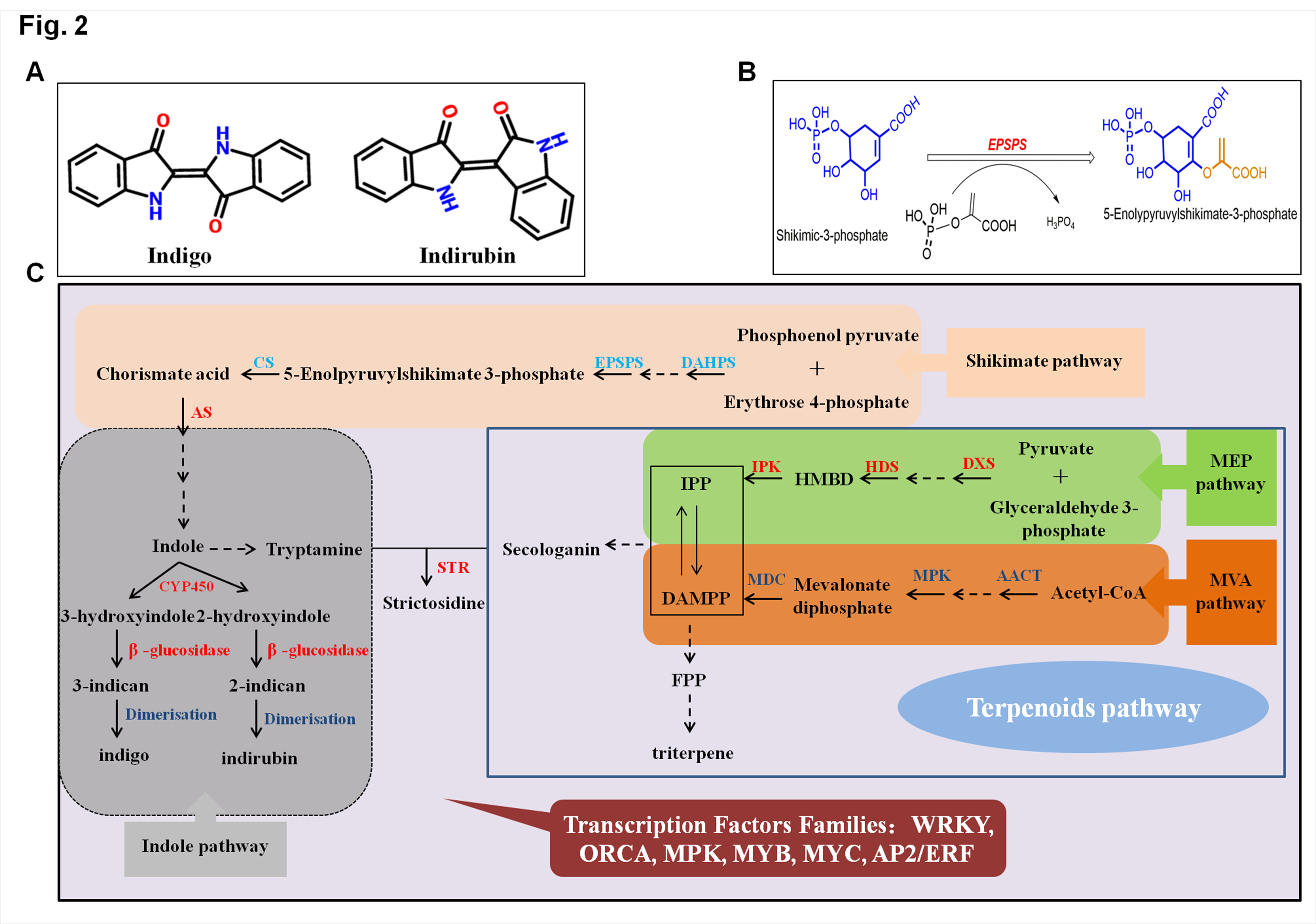

Supplement: Supplementary file 3 — Additional file 3: Figure S2. Prediction of the biosynthetic pathway of effective components in B. cusia and the catalytic reaction of EPSPS. (A) The chemical structure of indigo and indirubin. (B) The catalytic reaction of EPSPS. (C) The pink area is the Shikimate pathway. DAHPS, 3-deoxy-D-arabino-heptulosonate 7-phosphate synthase. EPSPS, 5-enolpyruvylshikimate-3-phosphate synthase. CS, chorismate synthase. The grey part is the tryptophan and indole pathway. AS, anthranilate synthase. TSA, tryptophan synthase alpha. CYP450, cytochrome P450 monooxygenase. The green part is the mevalonate pathway. DXS, deoxy-D-xylulose-5-phosphate synthase. HDS, hydroxymethylbutenyl-4-diphosphate synthase. HMBD, 1-hydroxy-2-methyl-2(D)butenyl-4-diphosphate. IPK, isopentenyl pyrophosphate kinase. IPP, isopentenyl pyrophosphate. The orange part is the MEP/DOXP pathway. AACT, acetoacetyl coenzyme thiolase. MPK, mevalonate phosphate kinase. MDC, mevalonate diphosphate decarboxylase. STR, strictosidine synthase. [file 12870_2019_2035_MOESM3_ESM.tif]

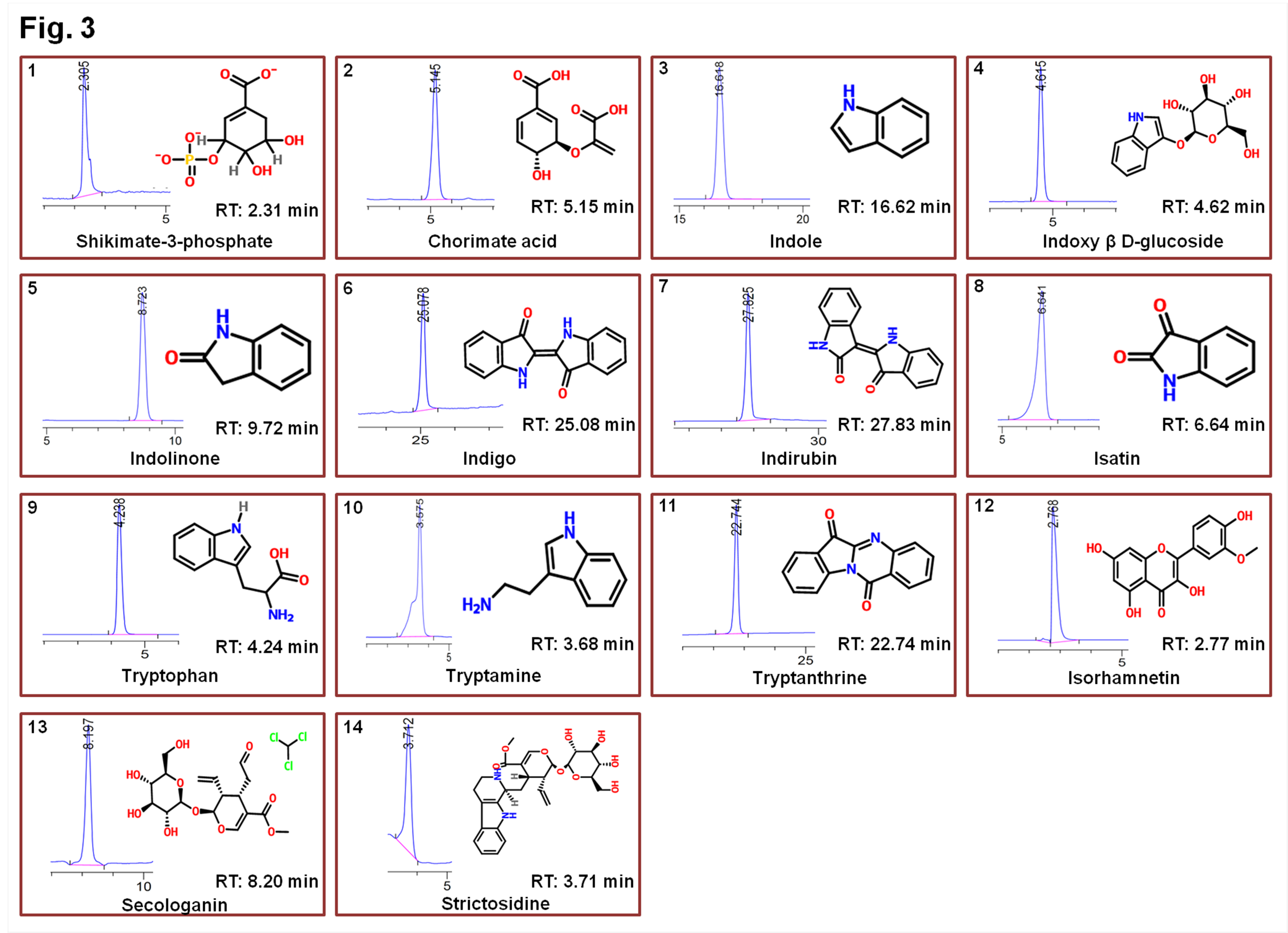

Supplement: Supplementary file 4 — Additional file 4: Figure S3. Chromatograms of 14 chemical compounds. The chromatograms with corresponding retention times of 14 chemical compounds, shikimate-3-phosphate, chorismic acid, indole, indoxyl beta D-glucoside, indolinone, indigo, indirubin, isatin, tryptophan, tryptamine, trytanthrin, isorhamnetin, secologanin and strictosidine. [file 12870_2019_2035_MOESM4_ESM.tif]

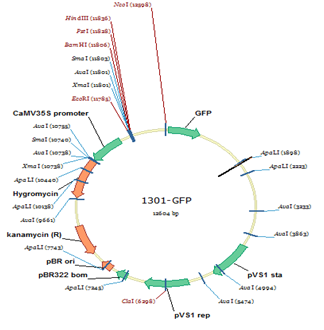

Supplement: Supplementary file 6 — Additional file 6. The pCAMBIA 1301-GFP vector [file 12870_2019_2035_MOESM6_ESM.tif]

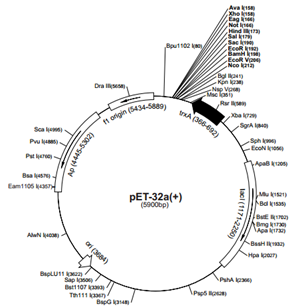

Supplement: Supplementary file 7 — Additional file 7. The pET 32a vector for constructing the fusion protein BcEPSPS-His. [file 12870_2019_2035_MOESM7_ESM.tif]

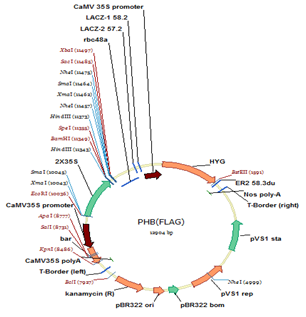

Supplement: Supplementary file 8 — Additional file 8. The overexpression vector PHB-flag. [file 12870_2019_2035_MOESM8_ESM.tif]
